# Lipid Vesicle Aggregation Induced by Cooling

**DOI:** 10.3390/ijms11020754

**Published:** 2010-02-21

**Authors:** Frank B. Howard, Ira W. Levin

**Affiliations:** Laboratory of Chemical Physics, National Institute of Diabetes and Digestive and Kidney Diseases, National Institutes of Health, Bethesda, Maryland 20892, USA; E-Mail: iwl@helix.nih.gov (I.W.L.)

**Keywords:** 1,2-dimyristoyl-sn-glycero-3-phosphocholine (DMPC), 1,2-dipalmitoyl-sn-glycero-3-phosphocholine (DPPC), 2-distearoyl-sn-glycero-3-phosphocholine (DSPC), 1,2-dipalmitoyl-sn-glycero-3-phosphoserine (DPPS), aggregation, single shell vesicles, lipid head groups

## Abstract

Lipid bilayer fusion is a complex process requiring several intermediate steps. Initially, the two bilayers are brought into close contact following removal of intervening water layers and overcoming electrostatic repulsions between opposing bilayer head groups. In this study we monitor by light scattering the reversible aggregation of phosphatidylcholine single shell vesicles during which adhesion occurs but stops prior to a fusion process. Light scattering measurements of dimyristoyl-sn-glycero-3-phosphocholine (DMPC), dipalmitoyl-sn-glycero-3-phosphocholine (DPPC) and 1,2-distearoyl-sn-glycero-3-phosphocholine (DSPC) in water show that lowering the temperature of about 0.14 micron single shell vesicles of DPPC (from 20 °C to 5 °C) and about 2 micron vesicles of DSPC (from 20 °C to 15 °C), but not of 1 micron vesicles of DMPC, results in extensive aggregation within 24 hours that is reversible by an increase in temperature. Aggregation of DSPC vesicles was confirmed by direct visual observation. Orientation of lipid head groups parallel to the plane of the bilayer and consequent reduction of the negative surface charge can account for the ability of DPPC and DSPC vesicles to aggregate. Retention of negatively charged phosphates on the surface and the burial of positively charged cholines within the bilayer offer an explanation for the failure of DMPC vesicles to aggregate. Lowering the temperature of 1,2-dipalmitoyl-sn-glycero-3-phosphoserine (DPPS) vesicles from 20 °C to 5 °C failed to increase aggregation within 24 hours at Mg^++^/DPPS ratios that begin to initiate aggregation and fusion.

## 1. Introduction

For eukaryotic cells to function properly, an efficient mechanism must exist for transferring macromolecular components between the internal cellular compartments or to and from the plasma membrane. Components packaged into lipid vesicles provide suitable vehicles for this essential function. For the transfer to occur, the vesicle and the target membrane must undergo a self assembly process requiring catalysis by special proteins. Included on the surface of the transport vesicle are specific recognition (v-SNARE) proteins [1,2] responsible for identifying the compartment of the vesicle’s origin and the class of cargo it carries. Acceptor membranes have complementary recognition proteins (t-SNARE) that pair with v-SNARE proteins both to ensure correct delivery of vesicle contents and to join the two proteins into a docking complex such that fusion can occur, thus completing self assembly. For the fusion process to take place, however, the energetically unfavorable displacement of the bound water layer on each membrane surface must be overcome, which requires the intervention of specific fusion proteins. While recognition proteins are essential for the biological docking function, we have noted that unmodified vesicles can aggregate without fusion in a process similar to docking in the absence of any proteins. The necessity for fusion protein intervention is clear, however, since spontaneous docking is not rapid enough to satisfy biological requirements.

In the absence of intervening proteins, membrane fusion is a multi-step, complex process [3–5]. That is, prior to fusion, the two bilayers must be brought into close contact, which can only occur after intervening bound water layers have been displaced and the electrostatic repulsions between the opposing membranes have been overcome. Although the mechanism of fusion with its several intermediate steps has been intensively studied [6–11], less attention has been paid to the initiation of the process; namely, the manner in which close contact occurs. In this manuscript, we focus on the reversible aggregation of small phosphatidylcholine single shell vesicles, in which close contact between bilayers leads to adhesion of the vesicles but not to fusion.

Aggregation of lipid vesicles composed of phosphatidylcholines, which have low negative surface charge densities, takes place more readily than vesicles of phosphatidylserines or phosphatidylethanolamines which exhibit greater negative surface charges. Phosphatidylcholines of mixtures containing both saturated and unsaturated lipid chains formed by extrusion into vesicles of a size measured at 25 °C to be about 120 nm [12] increased slowly to about 170 nm in 7 days and to a much larger irreversible size after 1 week. After 1 day, however, the vesicle size remained nearly unchanged. We are interested in observing the effect of temperature on aggregation of saturated lipid chain phosphatidylcholine vesicles, 1,2-dimyristoyl-sn-glycero-3-phosphocholine (DMPC), 1,2-dipalmitoyl-sn-glycero-3-phosphocholine (DPPC) and 1,2-distearoyl-sn-glycero-3-phosphocholine (DSPC), and have found that lowering the temperature of the single shell vesicles of DPPC and DSPC in water, but not DMPC, results in extensive aggregation without fusion within 24 hours. In contrast, we find that lowering the temperature of 1,2-dipalmitoyl-sn-glycero-3-phosphoserine (DPPS) vesicles does not affect the size of the particles even if nearly enough Mg^++^ is present to cause a large increase in size owing to screening of the electrostatic repulsion between the vesicles.

## 2. Results and Discussion

**Figure 1.**
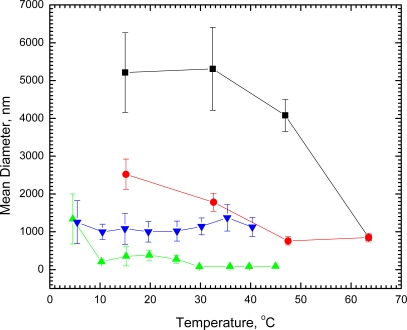
Dependence of mean diameter of vesicles on temperature. Inverted triangles, DMPC, heating; triangles, DPPC, heating; squares, DSPC, heating; circles, DSPC, cooling. Error bars indicate standard deviation of means.

We have found by laser light scattering measurements that dilute (1.26 × 10^−5^ M) single shell vesicles of DSPC in water 1,790 ± 230 nm in diameter undergo no significant change in size at room temperature within 41 days. In marked contrast, when DSPC vesicles of this size were cooled at 15 °C for 23 hours, the diameter increased nearly 3-fold to about 5,200 nm. Direct microscope observation of the DSPC vesicles at room temperature showed single unaggregated spheres. After being held overnight at 5 °C, visual observation revealed that the spheres aggregated into twos and threes without fusion, thus confirming the light scattering measurements. The diameters of the aggregated vesicles decreased with increasing temperature (Figure 1); and after the vesicles had undergone the gel to liquid crystal phase transition at 54 °C, the diameters were reduced markedly to only 850 nm at 63.5 °C. When cooled to 33 °C, the original diameter (1780 ± 240 nm) before any cooling was regained. On further cooling, the vesicles increased in diameter again; and, if held overnight at 15 °C, the diameters returned to the value found for the aggregated vesicles. A second cycle of heating and cooling retraced the curve for the DSPC vesicles (Figure 1). Undergoing the phase transition, thus, appears to have had no effect on the ability of the vesicles to aggregate again when exposed to reduced temperature.

We have also observed aggregation of vesicles with about a 10-fold smaller diameter. When vesicles of 1.42 × 10^−5^ M DPPC measured at 20.5 °C to be 135 ± 35 nm in diameter were cooled to 4.6 °C, within 2 hours the diameters increased 10-fold (to 1,340 ± 660 nm), indicating extensive aggregation, which was reversed with increasing temperature (Figure 1). Even at 10 °C, the vesicles decreased to about the original size (210 ± 55 nm) before cooling. Had either the DSPC or DPPC vesicles undergone fusion instead of aggregation, heating would not have reversed their status to produce particles of the same size and distribution that existed prior to cooling. In contrast, cooling vesicles of 1.48 × 10^−5^ M DMPC with a diameter at 14 °C of 1,100 ± 340 nm to 5.4 °C (diameter 1,250 ± 570 nm) was ineffective and showed no evidence of aggregation (Figure 1).

Although composed of neutral lipids, vesicles of DMPC, DPPC and DSPC exhibit a small net negative surface charge at low ionic strength [13], with DMPC vesicles having the largest negative charge and DSPC vesicles the smallest. The value of the surface charge is thought to reflect the orientation of the polar head group with respect to the bilayer plane, which varies with temperature, ionic strength and the length of the hydrocarbon chain [13]. At low ionic strengths and temperatures, the positively charged choline ends of the head groups of DMPC vesicles are buried within the interior of the bilayer, while the negatively charged phosphate groups occupy surface areas. With increasing temperature, the head groups reorient somewhat, with the cholines remaining below the surface while the phosphates remain at the surface. The choline ends of the head groups of DSPC vesicles, in contrast, are closer to the surface of the bilayer [13] and with increasing temperature, the head groups become parallel to the plane of the surface, abolishing the negative surface charge. With further increases in temperature, the cholines emerge from the surface while the phosphates sink below. Behavior of head groups of DPPC vesicles appear to be intermediate between that of DMPC and DSPC vesicles. Movement of head groups to a near parallel orientation to the bilayer surface and consequent reduction in the net negative surface charge offers an explanation for the ability of attractive van der Waals forces to overcome any residual electrostatic repulsion, allowing DPPC and DSPC vesicles to aggregate. Attractive interaction between opposite charges of parallel head groups of interacting vesicles may also contribute to aggregation. Failure of head groups to orient parallel to the bilayer surface and the consequent retention of a negative surface charge accounts for the failure to observe aggregation of DMPC vesicles. It is possible that charge presentation on a vesicle surface may be affected by the curvature of the bilayer. Marked curvature effects, however, apply to vesicles with smaller diameters than those studied here [14]. Curvature in small vesicles is thought to be large enough to affect the packing of phospholipid hydrocarbon chains resulting in a change in physical properties. Curvature in vesicles of the size we use, however, is too small to affect such packing because the surface is approximately planar and perpendicular with respect to the hydrocarbon chains. Our DMPC, DPPC and DSPC vesicles all have diameters sufficiently large that their surfaces are essentially planar, and any slight difference in curvature would be too small to account for the effects we observe. Other factors that may contribute to the failure to observe aggregation of DMPC vesicles at low temperature are a possible difference in the energy of displacement of intervening bound water layers and a difference in the attractive dispersion energy between potentially interacting bilayers.

Previous reports of the aggregation or fusion of DMPC, DPPC and DSPC vesicles differ in important ways from our results [14–17]. Most of the earlier observations were made on small vesicles which are unstable and fuse spontaneously and irreversibly to larger sizes. Our measurements were made on vesicles of significantly greater diameter, and the low-temperature aggregation detected was reversible. Aggregation and fusion of vesicles, moreover, can be expected to depend on concentration. The concentrations of vesicles used in our light scattering measurements were as much as three orders of magnitude smaller than those reported previously [14–17]. The low concentration of our vesicles appears to make any tendency to aggregate or fuse at room temperature or above a very slow process, much slower than the time required to observe an increase in diameter induced by low temperature.

**Figure 2.**
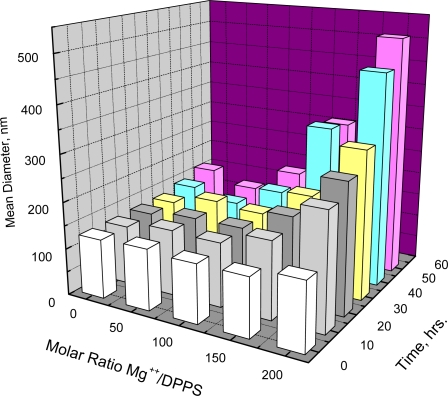
Aggregation at 20 °C of 1.29 × 10^−5^ M DPPS vesicles (137 ± 3 nm mean diameter) as a function of the molar ratio of Mg^++^/DPPS and of time.

In marked contrast to the aggregation observed for DPPC and DSPC is the failure to detect aggregation of DPPS at low temperature. Aggregation, however, is known to occur if the electrostatic repulsion between negatively charged DPPS vesicles is overcome by introduction of positively charged ions like Mg^++^ [18,19]. Figure 2 shows that such aggregation depends on both the molar ratio of Mg^++^/DPPS and time. When the Mg^++^/DPPS ratio was 20 or 50 there was no increase in mean diameter in less than 60 hours and only a slight increase at the Mg^++^/DPPS ratio equal to 100, but an increase was detectable within 2 hours at the Mg^++^/DPPS ratio equal to 200. Lowering the temperature to 5 °C at Mg^++^/DPPS ratios of 50, 100 or even 200, where the effect of electrostatic repulsion between negatively charged DPPS vesicles may be significantly reduced, however, resulted in no further increase in diameter within 24 hours. Thus, lowering the temperature at a Mg^++^/DPPS ratio just below the critical point where electrostatic repulsion between negatively charged DPPS vesicles would be largely reduced did not promote aggregation. Unlike the behavior of DPPC or DSPC, therefore, aggregation DPPS appears not to be affected by decreasing the temperature.

## 3. Experimental

### 3.1. Chemicals

DMPC, DPPC, DSPC and DPPS were supplied by Avanti Polar Lipids, Inc., Alabaster, Alabama, USA.

### 3.2. Preparation of Single-Shell Vesicles

About 0.1 g each of solid DMPC, DPPC, DSPC or 0.01 g of DPPS was transferred to separate 15 mL centrifuge tubes and weighed with a Sartorius semimicro analytical balance. Water was added to produce about a 25 weight percent lipid-water mixture, which was vibrated vigorously. The mixture was heated to 8–10 °C above the phase transition of the lipid for 1 minute, agitated for 30 seconds and placed in an ice bath for 30 seconds. The process was repeated nine times. Additional water was added to reduce the lipid content to 1 weight percent. The suspension was heated again (see above) and passed ten times through a 1 micron pore (DMPC and DSPC) Nucleopore Track-Etch Membrane (Whatman) held in place in a similarly heated Avanti extruder, producing single-shell vesicles. DPPC was passed successively through 1, 0.4, 0.2, 0.1, 0.08 and 0.05 micron pore membranes and DPPS through 0.4, 0.2 and 0.1 micron pore membranes.

### 3.3. Laser Light Scattering

Into 1 mL samples of 1:1,000 dilutions of 1 weight percent lipid vesicle suspensions of DMPC, DPPC or DSPC in a 10 × 75 mm test tube was inserted an Omega Type T thermocouple, held in place out of the path of the light beam with a Parafilm covered cork. Temperature was measured with an Omega Microcomputer Thermometer, Model HH-73T. The tube containing the sample was inserted into the thermostatable cell holder of a Brookhaven Instruments goniometer and illuminated with 514 nm green light from a Lexel Model 95 Ion Laser. Temperature was controlled with a Neslab Model RTE-100 refrigerated circulator. At each temperature, 10 measurements were made each of 2 minutes duration of the mean diameter of lipid vesicles. Data were processed with the Dynamic Light Scattering software from Brookhaven Instruments using both CONTIN and NNLS algorithms. The algorithms calculated closely similar results. Values from the CONTIN algorithm are shown in Figure 1. Differences in mean diameter as a function of temperature were determined with the Bonferroni multiple comparison test of the 1-way analysis of variance routine of the statistics program NCSS97 when variances were found to be equal by the Variance-Ratio Equal-Variance Test and by the Modified-Levene Equal-Variance Test. Differences in diameter when variances were found to be unequal were determined with the Two-Sample t-Test using the Aspin-Welch Unequal-Variance Test. Comparisons were made with the Kruskal-Wallis Z-value Test when distributions were found to be not normal. Mean diameters as a function of temperature are shown in Figure 1 together with standard deviations drawn as error bars.

Increasing Mg^++^/DPPS ratios in disposable light scattering cells (Malvern) containing 0.7 mL volumes of 1.29 × 10^−5^ M DPPS vesicles were prepared by adding appropriate volumes of more concentrated solutions of MgCl_2_. Mean diameters as a function of time (3 repeats per measurement) were measured at 20 °C with a Malvern Zeta Sizer light scattering apparatus. Diameters were determined with the Malvern Zetasizer software. Temperature was controlled with the instrument’s thermoelectric cell holder. Mean diameters as a function of both time and Mg^++^/DPPS ratio are shown in Figure 2. At Mg^++^/DPPS ratios of 50, 100 and 200 mean diameters over a 24-hour period were measured at 1 hr. intervals at 5 °C.

## 4. Conclusions

Our results indicate that lipid vesicles with low surface charge density like those composed of DPPC or DSPC can aggregate at low temperature but not rapidly fuse. The vesicles appear to approach each other in a process resembling docking and form an intermediate complex of sufficient stability that it may be detected by light scattering measurements. The structure of the contact region between vesicles is unknown, but the contact region must be large enough and sufficiently stable to permit experimental observation. It is through an understanding of initial bilayer adhesion steps that the complex fusion processes can ultimately be better illuminated.

## References

[1] Weber T Zemelman BV McNew JA Westermann B Gmachi M Parlati F Söllner TH Rothman JE SNAREpins: Minimal machinery for membrane fusion Cell 1998 92 759 772 9529252 10.1016/s0092-8674(00)81404-x

[2] Pelham HRP SNAREs and the specificity of membrane fusion Trends Cell. Biol 2001 11 99 101 11306253 10.1016/s0962-8924(01)01929-8

[3] Jahn R Südhof TC Membrane fusion and exocytosis Ann. Rev. Biochem 1999 68 863 911 10872468 10.1146/annurev.biochem.68.1.863

[4] Jahn R Grubmüller H Membrane fusion Curr. Opin. Cell Biol 2002 14 488 495 12383801 10.1016/s0955-0674(02)00356-3

[5] Jahn R Lang T Südhof TC Membrane fusion Cell 2003 112 519 533 12600315 10.1016/s0092-8674(03)00112-0

[6] Marrink SJ Eindahl E Edholm O Mark AE Simulation of the spontaneous aggregation of phospholipids into bilayers J. Am. Chem. Soc 2001 123 8638 8639 11525689 10.1021/ja0159618

[7] Noguchi H Takasu M Fusion pathways of vesicles: A brownian dynamics simulation J. Chem. Phys 2001 115 9547 9551

[8] Kozlovsky Y Kozlov M Stalk model of membrane fusion: Solution of energy crisis Biophys. J 2002 82 882 895 11806930 10.1016/S0006-3495(02)75450-7 PMC1301897

[9] Müller M Katsov K Schick M New mechanism of membrane fusion J. Chem. Phys 2002 116 2342 2345

[10] Markin VS Albanesi JP Membrane fusion: Stalk model revisited Biophys. J 2002 82 693 712 11806912 10.1016/S0006-3495(02)75432-5 PMC1301879

[11] Chernomordik LV Kozlov MM Membrane hemifusion: Crossing a chasm in two leaps Cell 2005 123 375 382 16269330 10.1016/j.cell.2005.10.015

[12] Roy MT Gallardo M Estelrich J Influence of size on electrokinetic behavior of phosphatidylserine and phosphatidylethanolamine lipid vesicles J. Colloid Interface Sci 1998 206 512 517 9756663 10.1006/jcis.1998.5715

[13] Makino M Yamada T Kimura M Oka T Ohshima H Kondo T Temperature- and ionic strength-induced conformational changes in the lipid head group region of liposomes as suggested by zeta potential data Biophys. Chem 1991 41 175 183 1773010 10.1016/0301-4622(91)80017-l

[14] Lichtenberg D Freire E Schmidt CF Barenholz Y Felgner PL Thompson TE Effect of surface curvature on stability, thermodynamic behavior, and osmotic activity of dipalmitoylphosphatidylcholine single lamellar vesicles Biochemistry 1981 20 3462 3467 6894860 10.1021/bi00515a024

[15] Larrabee AL Time-dependent changes in the size distribution of distearoylphosphatidylcholine vesicles Biochemistry 1979 18 3321 3326 465471 10.1021/bi00582a019

[16] Schullery SE Schmidt CF Felgner P Tillack TW Thompson TE Fusion of dipalmitoylphosphatidylcholine vesicles Biochemistry 1980 19 3919 3923 6893276 10.1021/bi00558a005

[17] van Dijck PWM de Kruijff B Aarts PAMM Verkleij AJ de Gier J Phase transitions in phospholipid model membranes of different curvature Biochim. Biophys. Acta 1978 506 183 191 620027 10.1016/0005-2736(78)90389-9

[18] Wilschut J Duzgunes N Papahadjopoulos D Calcium/magnesium specificity in membrane fusion: Kinetics of aggregation and fusion of phosphatidylserine vesicles and the role of bilayer curvature Biochemistry 1981 20 3126 3133 7248275 10.1021/bi00514a022

[19] Nir S Bentz J Wilschut J Mass action kinetics of phosphatidylserine vesicle fusion as monitored by coalescence of internal vesicle volumes Biochemistry 1980 19 6030 6036 7470447 10.1021/bi00567a013

